# Supplementary material for: Discovery and Validation of a Novel Step Catalyzed by OsF3H in the Flavonoid Biosynthesis Pathway
Source: Biology (Basel). 2021 Jan 6;10(1):32. doi: 10.3390/biology10010032 (PMC7825110; doi:10.3390/biology10010032)
Supplement: Supplementary file 1 [file biology-10-00032-s001.pdf]

Article

Discovery and Validation of a Novel Step Catalyzed by *OsF3H* in the Flavonoid Biosynthesis Pathway

Rahmatullah Jan[1], Sajjad Asaf[2], Sanjita Paudel[3], Lubna Lubna[4], Sangkyu Lee[3] and Kyung-Min Kim[1,*]

[1] Division of Plant Biosciences, School of Applied Biosciences, College of Agriculture & Life Science, Kyungpook National University, 80 Dahak-ro, Buk-gu, Daegu, 41566, Republic of Korea.
[2] Natural and Medical Science Research Center, University of Nizwa 616, Oman.
[3] College of Pharmacy, Research Institute of Pharmaceutical Sciences, Kyungpook National University, 80 Dahak-ro, Buk-gu, Daegu, 41566, Republic of Korea.
[4] Department of Botany, Garden Campus, Abdul Wali Khan University, Mardan 23200, Pakistan.
* Correspondence: kkm@knu.ac.kr; Tel.: +82-53-950-5711

Supplementary Material

Table S1. Solvents and conditions used for Kaempferol and quercetin extraction

System number	Chemical	Percentage	Condition and time	References
1	Ethanol	100	RT/1week	(Batubara et al., 2017)
2	Methanol	85	3hrs sonication	(Batubara et al., 2017)
3	Ethanol	70	70°C	(Batubara et al., 2017)

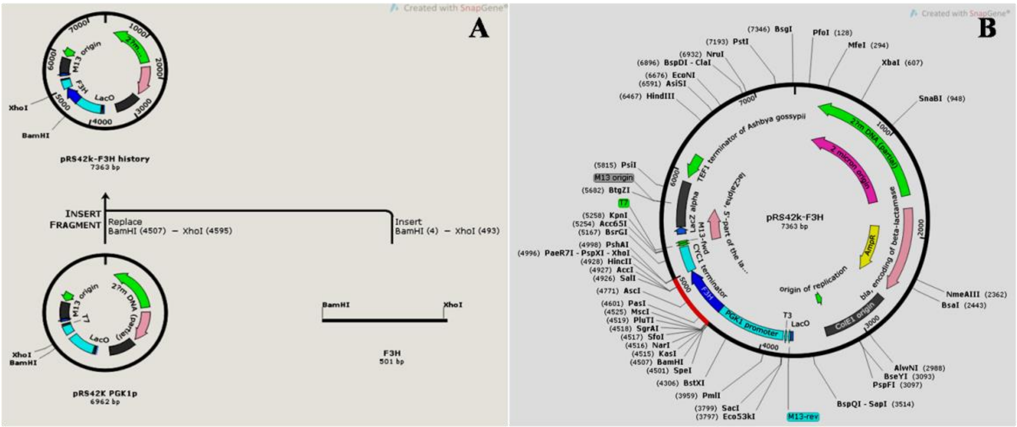

Figure S1. Diagrammatic representation of ligation. (**A**) history of ligation and (**B**) the construction of *OsF3H* expression vector.

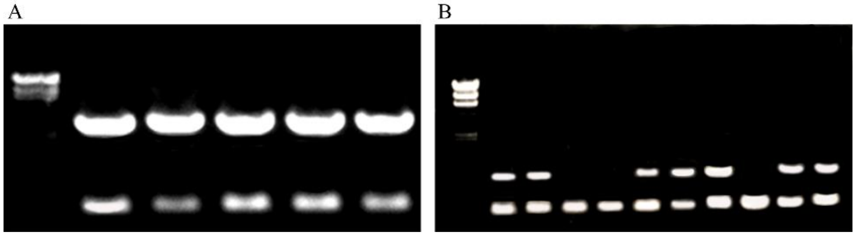

Figure S2. Cloning of *OsF3H* in yeast. (**A**)Colony PCR of *OsF3H* gene transformed to *S. cerevisiae* (D452-2) strain. (**B**) Transformation confirmation of *OsF3H* gene in *E.coli* cells using pRS42K yeast expression vector. The plasmid of five confirmed colonies were digested with BamH1 and Xho1 restriction enzymes, upper bands shows plasmid size while the lower indicates gene size.
